# Anatomical Atlas

**Published:** 1844-01

**Authors:** 


### ANATOMICAL ATLAS.

The medical profession in the United States, practitioners as well as students, will be obliged to Dr. Henry H. Smith for his beautiful Anatomical Atlas, the first part of which has been issued by the liberal publishers, Lea & Blanchard, of Philadelphia. The work is to be completed in five numbers, and will afford a most admirable illustration of the structure of the human body. That it is issued under the supervision of Professor Horner, to whose Special Anatomy and Histology it is to form the accompaniment, is an ample voucher for its fidelity and value. The original drawings are from the pencil of Pinkerton, who has acquired distinction in this department of art, and the engravings are in Gilbert's best style, which is the highest compliment that could be paid them. Of the utility of such drawings, the author truly says, there can be no queston. "Separated from the centre of instruction, and deprived of the advantages of the dissecting room, the ideas once so thoroughly acquired soon begin to fade, and the images once so distinct, become confused and mixed. A recourse to plates, in the absence of dead bodies, is then the only means of refreshing our knowledge."

It has been the aim of the author of the Atlas to comprise in it the valuable points of all previous works, to embrace the latest microscopical observations on the anatomy of the tissues, and, by placing it at a moderate price, to enable all to acquire it who may need its assistance in the dissecting or operating room, or other field of practice. The first part contains one hundred and thirty figures;—ninety-two of the bones, and thirty-six of the ligaments.

